# Neurofilament light chain in central nervous system infections: a prospective study of diagnostic accuracy

**DOI:** 10.1038/s41598-022-17643-9

**Published:** 2022-08-19

**Authors:** Ingeborg E. van Zeggeren, Liora ter Horst, Hans Heijst, Charlotte E. Teunissen, Diederik van de Beek, Matthijs C. Brouwer

**Affiliations:** 1 Department of Neurology, Amsterdam UMC, Amsterdam Neuroscience, University of Amsterdam, Meibergdreef 9, 1105 AZ Amsterdam, The Netherlands; 2 Department of Clinical Chemistry, Neurochemistry Laboratory, Amsterdam Neuroscience, Amsterdam UMC, Vrije Universiteit Amsterdam, de Boelelaan 1117, Amsterdam, The Netherlands

**Keywords:** Biochemistry, Neuroscience, Biomarkers, Neurology

## Abstract

Diagnosing central nervous system (CNS) infections quickly is often difficult. Neurofilament light chain (NfL) is a component of the axonal cytoskeleton and identified as marker of neuronal damage in several CNS diseases. We evaluated the diagnostic accuracy of NfL for diagnosing CNS infections. We included patients from a prospective cohort of consecutive patients in whom a lumbar puncture was performed for suspected CNS infection in an academic hospital in The Netherlands. The index test was NfL in cerebrospinal fluid (CSF) and reference standard the final clinical diagnosis. Diagnostic accuracy was determined using the area-under-the-curve (AUC) with 95% confidence intervals (CI). The association of CSF NfL with clinical characteristics, diagnosis and outcome was evaluated. Between 2012 and 2015, 273 episodes in adults of which sufficient CSF was available were included. CNS infection was diagnosed in 26%(n = 70), CNS inflammatory disease in 7%(n = 20), systemic infection in 32%(n = 87), and other neurological disorders in 33%(n = 90). Median CSF NfL level was 593 pg/ml (IQR 249–1569) and did not discriminate between diagnostic categories or CNS infection subcategories. AUC for diagnosing any CNS infection compared to patients without CNS infections was 0.50 (95% CI 0.42–0.59). Patients presenting with an altered mental status had higher NfL levels compared to other patients. We concluded that NfL cannot discriminate between causes in patients suspected of CNS infections. High concentrations of NfL are associated with severe neurological disease and the prognostic value of NfL in patients with CNS infections should be investigated in future research.

## Introduction

Patients suspected of a central nervous system (CNS) infection often pose a diagnostic dilemma. In a substantial part (76%) of the patients initially suspected of a CNS infection another diagnosis is made, including systemic infections without CNS involvement, metabolic encephalopathies, epilepsy or inflammatory diseases of the CNS^1^. Clinical characteristics fail to differentiate between these causes and the best predictor of a CNS infection is the cerebrospinal fluid (CSF) leukocyte count, but its sensitivity and specificity are still insufficient^1,2^. CNS infection can be caused by a wide variety of pathogens including bacteria, viruses, tuberculosis and fungi. In these patients the outcome depends on early initiation of targeted treatment^3,4^. Cultures or polymerase chain reaction (PCR), however, remain negative in a substantial proportion of the clinically suspected patients, ranging from 35–42% in viral CNS infections and 4–50% in bacterial meningitis^1,5–7^.

Neurofilament light chain (NfL) is a component of the axonal cytoskeleton, of which low levels are being constitutively released from axons into CSF and blood^8^. Increased NfL concentration has been identified as marker of neuronal damage due to a variety of central nervous system diseases^9^. These include multiple sclerosis (MS), Alzheimer’s disease, frontotemporal dementia, amyotrophic lateral sclerosis, traumatic brain injury and atypical Parkinsonian disorders^9–11^. NfL has been suggested to be of value in diagnosing some of these CNS diseases, but has also shown value as serum marker for response to treatment and prognosis^9–11^. Few studies have described neurofilaments in patients with CNS infections, of which one showed higher CSF levels of neurofilament heavy in children with bacterial meningitis compared to controls^12^. A recent study from our group showed CSF NfL level in bacterial meningitis patients was associated with poor prognosis, and showed levels differed significantly between causative pathogens^13^. Two other studies showed higher serum and CSF levels in patients with varicella zoster virus (VZV) encephalitis compared to VZV meningitis^14,15^. CSF levels of NfL in HIV patients showed that elevated levels were mostly found in those with HIV-associated dementia, but the diagnostic accuracy has not been studied in the at risk population^16^. Finally, in the past two years the value of CSF NfL levels has been evaluated in patients with neurological complications of COVID19, showing elevated levels in some, but no diagnostic value in differentiation between healthy controls or between those neurological manifestations in of COVID19^17,18^.

Our objective was to determine the diagnostic accuracy of NfL for the diagnosis of CNS infections. We hypothesized that NfL might be increased in patients with CNS infections in general and could function as a diagnostic biomarker in patients suspected of CNS infections. We measured levels of NfL in CSF of consecutive patients with suspected CNS infections from a previously collected, prospective cohort and evaluated the diagnostic accuracy of NfL. Furthermore, we analyzed whether NfL was associated with clinical characteristics and outcome in these patients.

## Methods

### Patients and samples

To assess the diagnostic accuracy of CSF NfL, the index test, for CNS infections, we analyzed patients who had been prospectively included in a cohort study of diagnostic parameters in suspected CNS infections, of which methods have been described in detail previously^1^. In brief, all consecutive episodes of inpatients or patients presenting to the emergency department of the Academic Medical Center in Amsterdam, The Netherlands, were prospectively included if they were ≥ 16 years and underwent a lumbar puncture for the suspicion of a CNS infection. When patients had multiple episodes of suspected CNS infections during the study period, each episode was included as separate entry in the study. Exclusion criteria were a neurosurgical procedure or severe neurotrauma less than three months prior to the lumbar puncture, or a neurosurgical device in situ. Data on clinical presentation, ancillary investigations and outcome were collected. Patients were then divided into groups based on their final clinical diagnosis: CNS infections, CNS inflammation without infection, systemic infection without CNS involvement, non-infectious non-inflammatory neurological disorders, and other systemic disorders. CNS infections were then subdivided into three categories: bacterial meningitis, viral meningitis and other CNS infections. Episodes were considered to be a CNS infection if there was microbiological evidence of infection, or when two neurologists independently classified the episodes as being due to a bacterial, viral or other CNS infection based on all available clinical data. Disagreements on the final diagnosis between the two neurologists were resolved by discussion with a third neurologist (kappa 0.76). This final diagnosis was considered the reference standard, to reflect clinical practice.

For this study, only episodes of patients with a sufficient amount of CSF available for NfL measurement (5 µl), the index test, were included. The CSF obtained during the first lumbar puncture at presentation or during admission was used. This was centrifuged after withdrawal and, after performance of regular diagnostics, frozen and stored in − 80 °C.

### NfL measuremens: Simoa

The index test, NfL, was measured in 5 µl of CSF using Simoa NF-light Advantage Kit (ref. 103186) on a HD-X instrument (Quanterix, Massachusetts, USA) at the Neurochemistry Laboratory at Amsterdam UMC, location VUmc, according to the manufacturer’s instructions. The investigators performing the NfL measurements were blinded to the reference standard.

### Statistical analysis

Statistical analysis was done using IBM SPSS Statistics for Windows, version 26 (Armonk, NY: IBM Corp.). Values are displayed as median with interquartile range (IQR) or absolute number with percentage. Continuous variables were compared by using the two-sample t-test or Mann–Whitney U test, depending on the distribution. Categorical data were compared using a Chi-square or Fisher’s exact test, depending on sample size. A p-value < 0.05 was considered statistically significant. We performed logistic regression analysis to determine the predictive value of NfL concentration in CSF with correction for age because of previous studies reporting an association of age and NfL levels in CSF. The area under the curve (AUC) of the receiver operating characteristic (ROC) curve was calculated to evaluate the diagnostic accuracy of NfL, with 95% confidence intervals (CI). There were no missing data on index and reference standard. As no prior data of the test characteristic of the index tests was available, no power calculation could be performed and the study is considered an exploratory diagnostic accuracy study. This study was reported according to the STARD criteria (Supplementary Table 1)^19^.

### Ethics approval

This study was approved by the Biobank Ethical Review Committee of the Amsterdam UMC (number METC 2014_290). Written informed consent was obtained from all participants or their representatives. All experiments were performed in accordance with relevant guidelines and regulations.

### Consent to participate

Written informed consent was obtained from all participants or their representatives.

## Results

**Figure 1:**
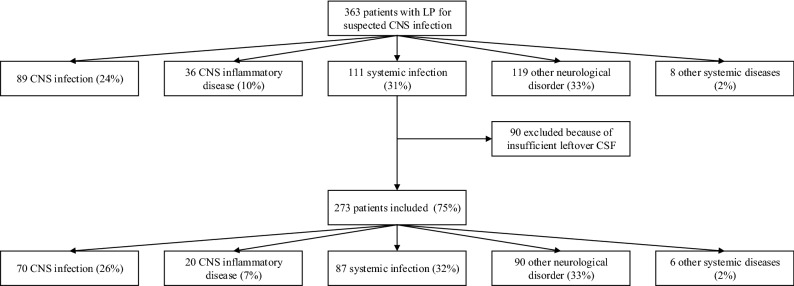
Inclusion flow chart.

Between 2012 and 2015, 363 episodes in 349 patients were included in the cohort with all consecutive episodes suspected of CNS infections (Fig. 1). In 273 episodes (75%) occurring in 264 patients, a sufficient amount of CSF was available for current analysis. Nine patients were included with two separate episodes of suspected CNS infections. The median age in these 273 episodes was 50 years (IQR 35–65) and 52% (n = 142) of the episodes occurred in women (Table 1). Overall median CSF leukocyte count was 4 × 10^6^/L (IQR 2–22) and median CSF total protein level was 0.4 g/L (IQR 0.3–0.7). A CNS infection was diagnosed in 26% (n = 70), CNS inflammatory disease in 7% (n = 20), systemic infection without CNS involvement in 32% (n = 87), non-infectious or -inflammatory neurological disorders in 33% (n = 90) and other systemic diseases in 2% (n = 6). Within the group of CNS infections, there were 15 episodes of bacterial CNS infection, 37 of viral CNS infection and 18 of other CNS infection (e.g. cryptococcal meningitis, tuberculous meningitis and cerebral toxoplasmosis). Mortality in all episodes was 11% (n = 30) and 26% (n = 72) had an unfavorable outcome, defined as a Glasgow Outcome Scale score of less than 5^20^.

**Table 1:** Baseline characteristics.

	All (n = 273)	CNS infection (n = 70)	CNS inflammatory disease (n = 20)	Systemic infection (n = 87)	Neurological disorder (n = 90)	Other systemic disease (n = 6)
Age (years)	50 (35–65)	45 (34–59)	50 (38–62)	52 (34–68)	53 (39–63)	53 (39–63)
Female sex	142 (52)	33 (47)	11 (55)	46 (53)	49 (54)	3 (50)
**Clinical presentation**						
Headache	159/266 (60)	46/67 (69)	16/19 (84)	55/84 (65)	40/90 (44)	2/6 (33)
Fever (≥ 38º)	106/271 (39)	31/70 (44)	1/19 (5)	52/87 (60)	21/89 (24)	1/6 (17)
Seizures	30/270 (11)	4/70 (6)	0/20 (0)	7/87 (8)	13/90 (14)	0/6 (0)
GCS score < 14	77/273 (28)	19/70 (27)	3/20 (15)	25/87 (29)	30/90 (33)	0/6 (0)
GCS score ≤ 8	25/273 (9)	5/70 (7)	0/20 (0)	7/87 (8)	13/90 (14)	0/6 (0)
Focal neurol. deficits	60/273 (22)	16/70 (23)	5/20 (25)	10/87 (11)	29/90 (32)	0/6 (0)
**CSF parameters**						
CSF leukocytes (10^6^/L)	4 (2–22)	147 (25–387)	16 (9–73)	4 (2–5)	4 (2–18)	3 (2–4)
CSF total protein (g/L)	0.4 (0.3–0.7)	0.8 (0.5–1.4)	0.7 (0.5–2.2)	0.3 (0.2–0.5)	0.4 (0.4–0.5)	0.4 (0.3–0.6)
**Outcome**						
Dead	30 (11)	9 (13)	2 (10)	8 (9)	10 (11)	1 (11)
Unfavorable*	72 (26)	19 (27)	5 (25)	17 (20)	30 (33)	1 (11)

Values are medians (interquartile range) or n/N (%). *CNS* central nervous system, *GCS* Glasgow Coma Scale, *CSF* cerebrospinal fluid.

*Unfavorable outcome is defined as a Glasgow Outcome Scale score of < 5.

**Figure 2:**
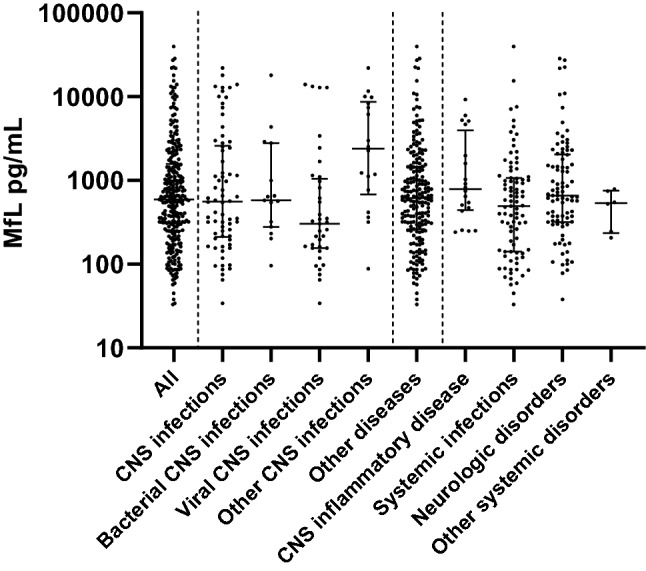
Concentration of NfL per diagnosis.

The median level of NfL was 593 pg/ml (IQR 249–1569) and did not differ between the different diagnostic categories (Kruskal–Wallis test, P = 0.44; Fig. 2; Supplementary Table 2). In episodes with CNS infections median NfL level was 558 pg/ml (IQR 212–2588) versus 615 pg/ml (IQR 263–1455) in other episodes (P = 0.70). Episodes of patients with bacterial meningitis had a median NfL level of 576 pg/ml (IQR 278–2777) compared to 603 pg/ml (IQR 248–1557; P = 0.99) in all other episodes, and compared to 303 pg/ml (IQR 156–1041; P = 0.22) in viral CNS infections. There were also no differences between groups after correction for age.

The AUC for diagnosing a CNS infection was 0.50 (95% CI 0.42–0.59). The AUC for diagnosing bacterial meningitis within all patients initially suspected of a CNS infection was 0.52 (95% CI 0.38–0.66). The AUC for differentiating bacterial meningitis from a viral CNS infection was 0.65 (95% CI 0.50–0.81).

**Figure 3:**
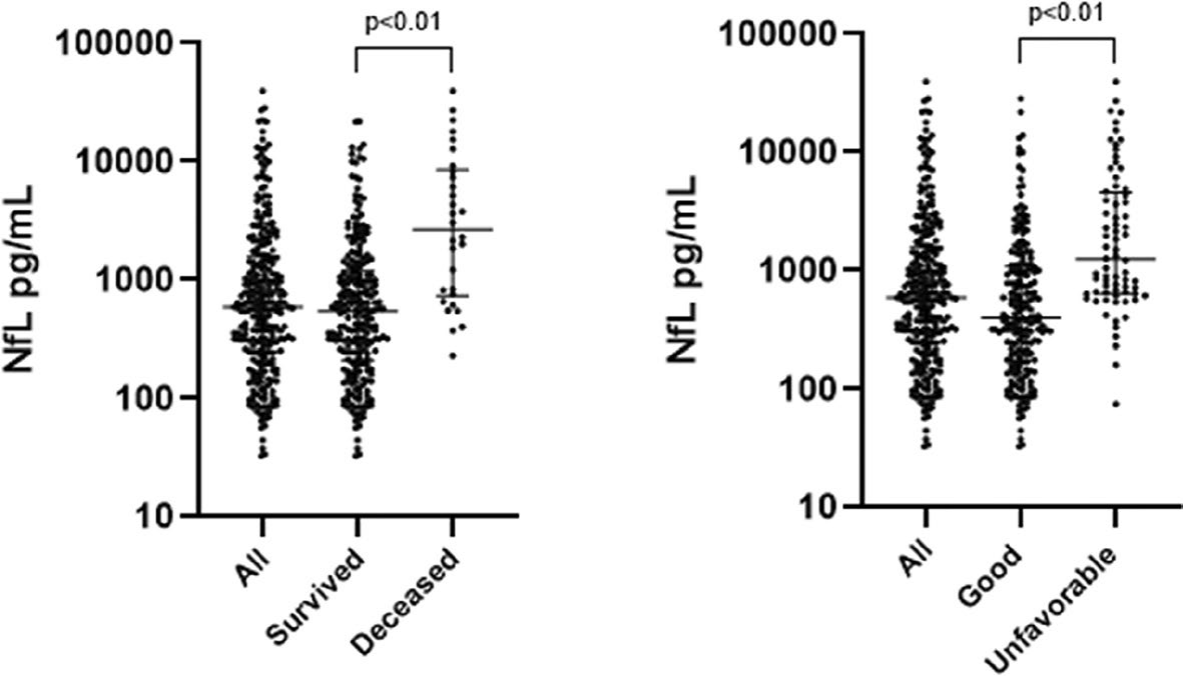
Concentration of NfL per outcome.

Episodes presenting with seizures had higher NfL levels (median 938 pg/ml [IQR 609–2422] vs. 547 pg/ml [IQR 218–1439]; P = 0.004), as did episodes presenting with focal neurological deficits (912 pg/ml [IQR 325–2525] vs. 556 pg/ml [IQR 206–1373]; P = 0.01). However, after correction for age, there was no association with seizure and focal deficits. In episodes in which the patient had an altered mental state, defined as a score on the Glasgow Coma Scale [GCS] < 14, NfL levels were significantly higher compared to patients with GCS scores of 14 or 15 (1051 pg/ml [IQR 590–2868] vs. 405 pg/ml [IQR196-1104]; P < 0.001). In comatose patients (GCS ≤ 8) this was 1996 pg/ml (IQR 641–4980) and 547 pg/ml (IQR 217–1384) in non-comatose patients (P = 0.001). This association remained significant after correction for age. A weak correlation between NfL and age was found (r = 0.5, P < 0.01) as well as between NfL and CSF protein (r = 0.4, P < 0.01). No correlation between NfL and sex or CSF leukocytes was found. Corrected for age, NfL was associated with mortality and unfavorable outcome (Fig. 3; odds ratio 1.16 [95% CI 1.08–1.24] and 1.14 [95% CI 1.06–1.22] per 1000 pg/ml).

## Discussion

Our study shows that the diagnostic accuracy for the diagnosis of CNS infections in all patients initially suspected of CNS infections is poor. No difference was found in levels of NfL in CSF between different diagnostic categories. NfL level in CSF was related to neurological symptoms, being associated with an altered mental status as well as seizures and focal neurological abnormalities, although the latter two were not significant after correction for age. Furthermore, higher levels of NfL were associated with mortality and unfavorable outcome.

In non-infectious neurological diseases, varying results have been reported on the diagnostic value of NfL^9,10^. The main limitation of these studies is the selection of patients to determine the diagnostic accuracy of NfL. In general these studies compared patients with the neurological disease to healthy controls, while this is not the population in which the differentiation needs to be made in clinical practice. For instance, in a study on diagnostic accuracy in Alzheimer’s disease, NfL in CSF was able to differentiate between one of the Alzheimer’s classification subgroups—patients with either tau pathology or neurodegeneration—and healthy controls, although only with an AUC of 0.69^21^. Differentiation between Alzheimer’s disease and frontotemporal dementia was poor, with an AUC of 0.54^21^. A meta-analysis of 15 retrospective studies showed that NfL was higher in patients with multiple sclerosis compared to healthy controls and could differentiate between these categories^22^. A good diagnostic accuracy was found for sporadic Creutzfeldt-Jakob’s disease (sCJD), although this was compared to a group with both psychiatric and a variety of non-neurodegenerative neurological diseases^23^. Differentiation between sCJD and several other neurodegenerative diseases was less accurate^23^. These findings are probably due to the fact that NfL is a very sensitive marker for axonal damage, but not specific. Therefore, it appears to discriminate best between neurological diseases with different degrees of axonal damage, rather than between disease categories^9,10^. Our study, including all patients suspected of CNS infections, does reflect clinical practice. However, the group of patients presenting with a suspected acute CNS infection probably is too heterogeneous to use CSF NfL as a diagnostic biomarker, since patients with other neurological diagnoses are also included.

We found NfL levels to be associated with an altered mental status in patients suspected of a CNS infection. This is consistent with NfL being a marker of axonal loss, which can be expected by generalized damage to the brain associated with this clinical characteristic. In several other neurological diseases such as multiple sclerosis and peripheral neuropathies NfL has been suggested to be helpful in monitoring disease activity and response to treatment^24,25^. This could especially be of help in diseases in which clinical evaluation is difficult and additional biomarkers are desirable that reflect the disease activity, like chronic auto-immune meningitis. A recent study in bacterial meningitis showed that NfL in bacterial meningitis was an independent predictor for unfavourable outcome, after correction for age, cranial nerve palsy, and high serum CRP levels^13^. This study also showed that CSF levels of Nfl correlated to the presence of an altered mental status and focal cerebral deficits, confirming CSF NfL levels reflect neuronal damage in CNS infections. In patients with suspected CNS infections, the prediction of outcome is however less informative, because a wide spectrum of diagnoses is still under consideration and NfL levels do not guide the differential diagnosis enabling targeted treatment^1^.

Our study has several limitations. First, the current analysis was a retrospective study on biobanked CSF samples. Clinical data, however, were collected prospectively and bias should therefore be limited. Second, we performed just one measurement of NfL level per patient, so the course of NfL in CSF of these patients was not evaluated. We did not have data on the exact date of onset of symptoms in our database and it might therefore be possible that patients reached higher levels of NfL later during the disease. For the diagnostic evaluation of NfL this would not matter, since the diagnosis is needed as soon as possible and preferably in the initial CSF sample. However, for prognostic purposes measuring several time-points could provide additional information^12^. Preferably, this would be done in blood samples instead of CSF because of the easier accessibility. The correlation between serum and CSF concentrations should, however, be evaluated in patients with CNS infections more thoroughly, since this correlation can vary in different disease types^24,26,27^. Finally, in only 75% of the patients from the prospectively collected cohort a sufficient amount of CSF was available. Since the distribution of diagnoses corresponds well to the original cohort, selection bias because of this is limited.

## Conclusion

In conclusion, NfL in CSF has a poor diagnostic accuracy in patients suspected of CNS infections. NfL is associated with clinical signs associated with damage to the nervous system disease and unfavourable outcome. The use of NfL for prognosis or therapy monitoring for CNS infections in patients with elevated levels should be investigated in future research.

## Supplementary Information

Supplementary Information.

## Data Availability

The datasets used and/or analysed during the current study are available from the corresponding author on reasonable request.

## Abbreviations

AUC: Area under the curve
CI: Confidence interval
CNS: Central nervous system
CSF: Cerebrospinal fluid
GCS: Glasgow Coma Scale
IQR: Interquartile range
MS: Multiple sclerosis
NfL: Neurofilament light chain
PCR: Polymerase chain reaction
ROC: Receiving operator curve
sCJD: Sporadic Creutzfeldt-Jakob’s disease
VZV: Varicella zoster virus
