# Supplementary material for: Neurofilament light chain in central nervous system infections: a prospective study of diagnostic accuracy
Source: Sci Rep. 2022 Aug 19;12:14140. doi: 10.1038/s41598-022-17643-9 (PMC9391449; doi:10.1038/s41598-022-17643-9)
Supplement: Supplementary file 1 — Supplementary Information. [file 41598_2022_17643_MOESM1_ESM.docx]

**SUPPLEMENTAL MATERIAL**

**Supplementary Table 1. STARD guideline checklist**

| Section and topic | No | Item | Page no. manuscript |
| --- | --- | --- | --- |
| Title or abstract |  |  |  |
|  | 1 | Identification as a study of diagnostic accuracy using at least one measure of accuracy (such as sensitivity,  specificity, predictive values, or AUC) | 1 |
| Abstract |  |  |  |
|  | 2 | Structured summary of study design, methods, results, and conclusions (for specific guidance, see STARD  for Abstracts) | 1 |
| Introduction |  |  |  |
|  | 3 | Scientific and clinical background, including the intended use and clinical role of the index test | 3 |
|  | 4 | Study objectives and hypotheses | 3 |
| Methods |  |  |  |
| Study design | 5 | Whether data collection was planned before the index test and reference standard were performed (prospective  study) or after (retrospective study) | 4 |
| Participants | 6 | Eligibility criteria | 4 |
|  | 7 | On what basis potentially eligible participants were identified (such as symptoms, results from previous tests,  inclusion in registry) | 4 |
|  | 8 | Where and when potentially eligible participants were identified (setting, location, and dates) | 4 |
|  | 9 | Whether participants formed a consecutive, random, or convenience series | 4 |
| Test methods | 10a | Index test, in sufficient detail to allow replication | 4 |
|  | 10b | Reference standard, in sufficient detail to allow replication | 4 |
|  | 11 | Rationale for choosing the reference standard (if alternatives exist) | 4 |
|  | 12a | Definition of and rationale for test positivity cut-offs or result categories of the index test, distinguishing  pre-specified from exploratory | NA |
|  | 12b | Definition of and rationale for test positivity cut-offs or result categories of the reference standard, distinguishing  pre-specified from exploratory | 4 |
|  | 13a | Whether clinical information and reference standard results were available to the performers or readers of  the index test | 4 |
|  | 13b | Whether clinical information and index test results were available to the assessors of the reference standard | 4 |
| Analysis | 14 | Methods for estimating or comparing measures of diagnostic accuracy | 5 |
|  | 15 | How indeterminate index test or reference standard results were handled | 5 |
|  | 16 | How missing data on the index test and reference standard were handled | 5 |
|  | 17 | Any analyses of variability in diagnostic accuracy, distinguishing pre-specified from exploratory | 5 |
|  | 18 | Intended sample size and how it was determined | 5 |
| Results |  |  |  |
| Participants | 19 | Flow of participants, using a diagram | Page 5; Figure 1 |
|  | 20 | Baseline demographic and clinical characteristics of participants | Page 5; Table 1 |
|  | 21a | Distribution of severity of disease in those with the target condition | Page 6; Table 1 |
|  | 21b | Distribution of alternative diagnoses in those without the target condition | Page 5; Figure 1 |
|  | 22 | Time interval and any clinical interventions between index test and reference standard | NA |
| Test results | 23 | Cross tabulation of the index test results (or their distribution) by the results of the reference standard | Page 6; Figure 2; Supplementary Table 2 |
|  | 24 | Estimates of diagnostic accuracy and their precision (such as 95% confidence intervals) | 6 |
|  | 25 | Any adverse events from performing the index test or the reference standard | NA |
| Discussion |  |  |  |
|  | 26 | Study limitations, including sources of potential bias, statistical uncertainty, and generalisability | 8 |
|  | 27 | Implications for practice, including the intended use and clinical role of the index test | 9 |
| Other information |  |  |  |
|  | 28 | Registration number and name of registry | 5 |
|  | 29 | Where the full study protocol can be accessed | NA |
|  | 30 | Sources of funding and other support; role of funders | 9 |

NA= not applicable

**Supplementary Table 2. NfL concentrations per subcategory**

| Diagnosis | Median NfL concentration (pg/ml) |
| --- | --- |
| **CNS infections (n=70)** | 558 (212-2588) |
| Bacterial meningitis (n=15) | 576 (278-2777) |
| Viral meningitis (n=25) | 196 (131-321) |
| Viral encephalitis (12) | 959 (400-10,474) |
| Progressive multifocal leucencephalopathy (n=3) | 7393 (NA) |
| Cryptococcal meningitis (n=3) | 2258 (NA) |
| Tuberculous meningitis (n=3) | 1114 (NA) |
| Parasitic encephalitis (n=2) | 5408 (NA) |
| Cerebral toxoplasmosis (n=2) | 11601 (NA) |
| Leptospirosis meningitis (n=2) | 251 (NA) |
| **CNS inflammatory disease (n=20)** | 783 (441-3980) |
| Auto-immune encephalitis (n=4) | 3310 (973-4994) |
| Chronic meningitis (n=5) | 912 (498-5130) |
| Recurrent aseptic meningitis (n=4) | 451 (295-526) |
| Neurosarcoidosis | 3113 (NA) |
| **Systemic infection (n=87)** | 491 (141-1070) |
| Respiratory tract infection (n=20) | 999 (423-3610) |
| Bacteremia/ systemic bacterial infection (n=15) | 402 (149-745) |
| Systemic viral infection (n=10) | 165 (69-450) |
| Ear, nose or throat infection (n=12) | 313 (96-791) |
| Gastro-intestinal infection (n=11) | 204 (86-651) |
| Genito-urinary tract infection (n=7) | 1628 (321-7562) |
| Skin/soft tissue infection (n=5) | 1020 (718-1942) |
| Fever or sepsis eci (n=4) | 345 (78-2803) |
| **Other neurological disease (n=90)** | 659 (319-2036) |
| Headache syndrome (n=29) | 373 (122-864) |
| Metabolic or toxic encephalopathy (n=21) | 761 (416-2575) |
| Epilepsy (n=14) | 934 (549-1596) |
| Stroke (n=11) | 1418 (658-4931) |
| Intracerebral tumor (n=6) | 1652 (443-9777) |
| Functional neurological symptoms (n=3) | 317 (NA) |
| **Other systemic disease (n=6)** | 536 (235-754) |
| Psychiatric condition (n=3) | 742 (NA) |
| Systemic auto-immune disease (n=2) | 536 (NA) |
| NfL= neurofilament light chain, CNS= central nervous system, NA= not applicable | |
